# Persistent hiccups due to aripiprazole: a case report and review of the literature

**DOI:** 10.3389/fphar.2023.1284510

**Published:** 2024-01-05

**Authors:** Yaoyin Zhang, Wei Chen, Junming Chen, Mingmei Li, Yulan Huang, Wenjiao Min

**Affiliations:** ^1^ Department of Psychosomatics, Sichuan Provincial Center for Mental Health, Sichuan Provincial People’s Hospital, School of Medicine, University of Electronic Science and Technology of China, Chengdu, China; ^2^ Key Laboratory of Psychosomatic Medicine, Chinese Academy of Medical Sciences, Chengdu, China; ^3^ Sichuan Provincial Center for Mental Health, Sichuan Academy of Medical Sciences and Sichuan Provincial People’s Hospital, Chengdu, China

**Keywords:** aripiprazole, hiccup, adverse effects, management, psychosocial factor

## Abstract

**Introduction:** Aripiprazole, a commonly prescribed antipsychotic, has been rarely associated with the onset of hiccups. This study aims to elucidate the prevalence, risk factors, and management of aripiprazole-induced hiccups.

**Methods:** We report a case of aripiprazole-induced hiccups in a 32-year-old male diagnosed with Somatic Symptom Disorder per DSM-5 criteria.A comprehensive literature review was conducted, identifying 29 case reports of aripiprazole-induced hiccups. Patient demographics, dosage, onset and duration of hiccups, and management strategies were analyzed.

**Results:** Aripiprazole-induced hiccups predominantly affected adolescents and middle-aged male patients (86.7%). The majority of hiccups developed within 1–2 days post-prescription (90.9%) and resolved within 1–4 days after discontinuation of aripiprazole. Discontinuation of aripiprazole was the most effective management strategy (51.7%). Co-administration with benzodiazepines was identified as a significant risk factor.

**Discussion:** The findings suggest that clinicians should be vigilant for the onset of hiccups during the early stages of aripiprazole treatment, especially in male patients and those co-administered with benzodiazepines.

**Conclusion:** Clinicians should be vigilant for hiccups during early aripiprazole treatment. Considering personality and psychological factors is crucial in managing hiccups in psychiatric patients.

## 1 Introduction

Aripiprazole, a third-generation antipsychotic, stands out due to its distinct pharmacodynamic characteristics. It functions as a partial agonist for both dopamine D2 and serotonin 5-HT1A receptors, and as an antagonist for the serotonin 5-HT2A receptor. It’s reported that aripiprazole has selective effects on upregulating the GABAA (β-1) receptor (Pan et al., 2016). Although it is generally well-tolerated with a favorable metabolic profile and a relatively limited array of side effects, some reports have associated it with persistent hiccups (Serafini et al., 2019). This article explores this uncommon adverse reaction and its broader implications.

**FIGURE 1:**
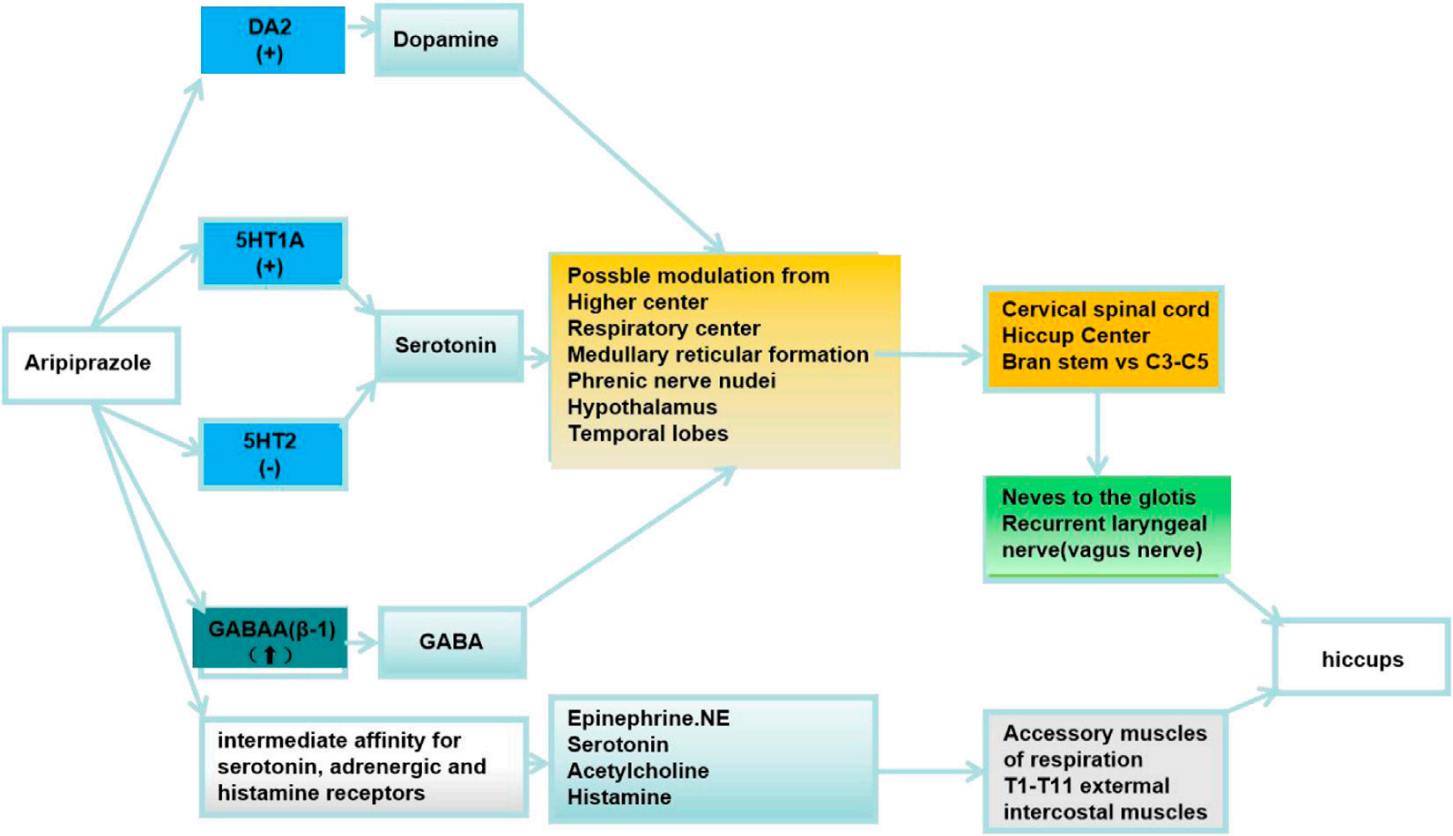
DA:Dopamine D2 receptors; 5HT1A:serotonin 5-HT1A receptors; 5HT2A:serotonin 5-HT2A receptors; GABAA (β-1):Gamma-aminobutyric acid A (β-1) receptor; (↑):upregulating the receptor; (+):partial agonist; (−):antagonist.

Hiccups are caused by involuntary spasms of the diaphragm, which lead to an abrupt inhalation and a rapid closure of the glottis, producing the distinctive hiccup sound. Typically, hiccups commence without a discernible cause and resolve spontaneously within a short duration. They are predominantly benign and self-terminating. Hiccups persisting for over 48 h are termed “persistent hiccups.” If the condition extends beyond 1 month, it is referred to as “intractable hiccups” (Nausheen et al., 2016). The underlying mechanism is thought to be the stimulation of the “hiccup reflex” arc. This reflex involves afferent signals from the phrenic, vagus, and sympathetic nerves (T6–T12) that relay to central processing areas in the cervical spine (C3-C5), brainstem, reticular activating system, and hypothalamus. The efferent signals are transmitted through motor fibers from the phrenic nerve to the diaphragm, the accessory nerve to the intercostal muscles, and the recurrent laryngeal nerve to the glottis. Central neurotransmitters implicated in hiccups include gamma-aminobutyric acid (GABA), dopamine, glutamine, and serotonin. Peripheral transmitters involved are epinephrine, norepinephrine, acetylcholine, and histamine. Various factors can induce hiccups, such as central nervous system tumors, inflammatory diseases, gastrointestinal disorders, specific medications, and psychological conditions. Furthermore, hiccups can also be initiated by other physical or chemical stimuli (Carbone et al., 2021). The association between aripiprazole administration and the onset of hiccups is depicted in Figure 1. This correlation was elucidated through a comprehensive review of the literature (Nausheen et al., 2016). However, the precise underlying mechanism governing this relationship remains to be elucidated and warrants further investigation.

In this study, we detail a case of hiccups induced by aripiprazole, intertwined with psychogenic elements, and offer an exhaustive review of the current literature on this subject.

### 1.1 Case report

The male patient, aged 32, was admitted to the hospital due to intensified health anxieties, accompanied by palpitations and a sensation of chest constriction that had persisted for over 5 years. He exhibited traits of impatience and anxiety, was highly suggestible, and had a dependent personality. He felt a profound lack of understanding and support from his wife and was torn about seeking a divorce after uncovering her infidelity 2 months earlier. Notably, his father had previously been diagnosed with an anxiety disorder. In alignment with the DSM-5 guidelines, the patient was diagnosed with Somatic Symptom Disorder. Furthermore, he had been living with chronic hepatitis B for 10 years, consistently adhering to a regimen of Tenofovir fumarate (25 mg daily), and his liver functions were within normal parameters.

The primary focus of the therapeutic intervention was to address the patient’s ambivalence towards his marital relationship. He expressed feelings of being misunderstood and uncared for by his wife, yet simultaneously demonstrated a deep dependency on her. Comprehensive evaluations conducted after his admission, encompassing blood tests, liver and kidney function tests, electrolyte levels, and electrocardiograms, did not indicate any significant abnormalities. His score on the Patient Health Questionnaire 15 (PHQ-15) was 6, suggesting mild physical symptoms. Meanwhile, his scores on the PHQ-9 and the Generalized Anxiety Disorder 7-item (GAD-7) were 11 and 14, respectively, pointing to moderate levels of depression and anxiety.

The initial treatment plan included escitalopram (20 mg daily) and oxazepam (67.5 mg daily). However, despite a week of this regimen, the patient continued to exhibit significant anxiety and an excessive preoccupation with his health. As a result, the treatment was adjusted: oxazepam was replaced with lorazepam (2.5 mg daily), and aripiprazole (5 mg daily, taken after dinner) was added. Notably, the patient began experiencing persistent hiccups roughly 6 h after the first dose of aripiprazole. Even with the administration of anisodamine (5 mg, three times daily) and baclofen (10mg, twice daily), the hiccups persisted without notable improvement. After the next dose of aripiprazole, the patient reported an exacerbation of the hiccups, which severely disrupted his sleep and appetite and heightened his anxiety levels. A dental examination ruled out any oral issues. Common remedies, such as holding his breath or pinching his nose, were ineffective. By the third night, the patient experienced almost total sleep deprivation. On the fourth day, despite receiving a 10 mg injection of metoclopramide, there was no improvement in his condition, and his anxiety markedly increased. That evening, the decision was made to discontinue aripiprazole and administer a 25 mg dose of chlorpromazine. This intervention provided relief from the hiccups and allowed the patient sporadic sleep. Remarkably, by the early hours of the fifth day, roughly 8 hours after the chlorpromazine dose and 33 h after discontinuing aripiprazole, the hiccups had completely subsided.

Following the episode, the patient was maintained on a regimen of escitalopram (20 mg daily) and lorazepam (1.5 mg daily). Subsequent follow-up visits revealed significant alleviation in his anxiety levels and palpitations. However, a hiccup recurrence was noted a month after his discharge. This episode was less severe, causing only minor disruptions to his daily activities and sleep patterns. Fortunately, the hiccups subsided after the patient underwent psychological suggestion therapy during an outpatient clinic session.

## 2 Discussion

### 2.1 Case characteristics

The patient, a young male exhibiting paranoid tendencies, suggestibility, and a dependent nature, had a longstanding history of chronic hepatitis B. Within hours of receiving a regimen of escitalopram (20 mg/d), lorazepam (2.5 mg/d), and aripiprazole (5 mg/d), he developed severe hiccups that profoundly impacted his emotional wellbeing and sleep quality. Despite the continued use of aripiprazole, the hiccups persisted, showing no improvement even after treatments with anisodamine (5 mg tid), baclofen (10 mg bid), or a metoclopramide injection (10 mg) (Steger et al., 2015). Relief was only achieved after discontinuing aripiprazole and administering an injection of chlorpromazine (25 mg) (National Pharmacopoeia Committee, 2020). The hiccups completely subsided 33 h post-aripiprazole discontinuation and 7 h after the chlorpromazine injection. Furthermore, no alternative antipsychotic medications were employed in this case. However, a month after his discharge, the patient experienced a brief recurrence of milder hiccups, which minimally impacted his daily activities and sleep. This episode was swiftly addressed and resolved during an outpatient psychological counseling session.

### 2.2 Literature review analysis

Methods: We conducted an exhaustive literature search across several databases, including PubMed, Embase, Cochrane Library, PsycINFO, Wanfang Data, and CNKI, covering the period from their inception to 18 February 2023. We utilized the search terms “hiccup,” “hiccough,” “singultus,” and “aripiprazole.” This search produced fourteen case or case series reports, one retrospective study, a single case report, and a literature review. In all, we included twenty-nine case reports that described hiccups triggered by aripiprazole in our review.

From our literature review, we identified the following clinical characteristics associated with aripiprazole-induced hiccups:

**Demographics:** Hiccups induced by aripiprazole are most prevalent among adolescent and middle-aged male patients, comprising 86.7% of the reported instances.

**Mental Disorders Association:** There’s no specific correlation between mental disorders and aripiprazole-induced hiccups. Such hiccups are prevalent across various mental conditions:

• Bipolar disorder: 41.4% (12/29)
• Schizophrenia and its spectrum disorders, as well as depression: 17.2% each (5/29)
• Substance abuse: 10.3% (3/29)
• A collective 13.8% (4/29) for obsessive-compulsive disorder, schizoaffective disorder, and neurodevelopmental disorder.

**Dosage Relationship:** The correlation between the onset of hiccups and the dosage of aripiprazole is not clearly defined. Dosages varied from 2.5 mg/d to 30 mg/d. Cases induced by lower dosages (2.5–10 mg) constituted 51.7%, whereas those resulting from medium to high dosages (11–30 mg) comprised 48.3%.

**Onset and Duration:** Most aripiprazole-induced hiccups (90.9%) appeared within 1–2 days of medication initiation. Generally, these hiccups resolved within 1–4 days after discontinuing aripiprazole. Notably, in older patients, the resolution period extended to between 4 and 7 days.

### 2.3 Intervention strategies

• The primary method to mitigate aripiprazole-induced hiccups, employed in 51.7% (15/29) of cases, was the cessation of aripiprazole.
• When aripiprazole was used in conjunction with benzodiazepines, stopping the benzodiazepines was the common strategy, proving effective in 24.1% (7/29) of cases.
• Gabapentin/pregabalin also emerged as an effective treatment, alleviating symptoms in 20.7% (6/29) of cases.
• After the hiccups subsided, the majority of patients discontinued aripiprazole. However, in a small subset (6.8%, 2/29), aripiprazole was maintained, with patients eventually tolerating and overcoming the hiccups.
• Transitioning from aripiprazole to other antipsychotic medications, such as risperidone, quetiapine, olanzapine, acenapine, or clozapine, successfully prevented hiccup recurrence in 41.3% (12/29) of instances.

#### 2.3.1 The vulnerable population

Aripiprazole-induced hiccups are most commonly observed in adolescent and middle-aged male patients, making up 86.7% of reported cases. A detailed age breakdown is as follows: adolescents (12–18 years) constitute 5 of the 29 cases; young adults (19–35 years) represent 15 of the 29 cases; middle-aged individuals (36–59 years) account for 6 of the 29 cases; and the elderly (60 years and above) comprise the remaining 3 cases. The youngest reported patient was 13 years old, while the oldest was 69 years old.

In prior research, 80% of intractable hiccups were observed in elderly men, a demographic distinct from those experiencing aripiprazole-induced hiccups. Aripiprazole is typically prescribed to adults and young adults, aligning with the age groups most commonly diagnosed with psychiatric disorders. Both aripiprazole-induced hiccups and intractable hiccups from other studies suggest that males are more susceptible to this condition than females. Our literature review corroborates this, revealing that out of 29 cases of aripiprazole-induced hiccups, 26 were in males and only 3 in females, which is consistent with previous findings (Souadjian and Cain, 1968).

#### 2.3.2 The relationship between mental disorder diagnoses and aripiprazole-induced hiccup

There appears to be no direct correlation between the onset of aripiprazole-induced hiccups and the specific diagnosis of mental disorders. Hiccups can emerge in patients with a variety of conditions, including neurodevelopmental disorders (such as intellectual disorders, ADHD, and conduct disorder), schizophrenia and its spectrum disorders, bipolar disorder, depression, and obsessive-compulsive disorder. To provide a breakdown: bipolar disorder, schizophrenia and its spectrum disorders, depression, substance abuse, and neurodevelopmental disorders represented 41.4% (12/29), 17.2% (5/29), 17.2% (5/29), 10.3% (3/29), and 13.8% (4/29) of cases, respectively. This study highlights a case of hiccups in a patient diagnosed with somatic symptom disorder. Thus, from our perspective, the onset of hiccups may be more directly linked to aripiprazole itself rather than the underlying mental condition, a conclusion that aligns with findings from prior case reviews (Serafini et al., 2019).

#### 2.3.3 Dosage and administration route in relation to aripiprazole-induced hiccup

The onset of aripiprazole-induced hiccups does not exhibit a consistent correlation with the drug’s dosage. Dosages in reported cases varied from 2.5 mg/d to 30 mg/d. Interestingly, hiccups induced by lower doses (2.5–10 mg) were slightly more common, accounting for 51.7% of cases, while those triggered by medium to high doses (11–30 mg) represented 48.3%. In our study, the patient experienced persistent hiccups even at a lower dose of aripiprazole (5 mg/day), which significantly disrupted sleep. To date, our extensive review of the literature reveals no substantial correlation between the dosage of aripiprazole and the incidence of hiccups, suggesting that dosage may not be a critical factor in the etiology of hiccups. This observation underscores the necessity for more detailed research within a controlled clinical setting. Furthermore, the route of aripiprazole administration does not seem to play a decisive role in the onset of hiccups. Of the cases reviewed, five patients were administered aripiprazole via intramuscular injection, thirteen received it orally, and the administration method for the remaining cases was not specified.

The exact pathways through which varying dosages of aripiprazole induce hiccups remain a topic of debate, with no consensus yet established. Current hypotheses center around aripiprazole’s modulation of both dopaminergic (Silverman et al., 2014) and serotonergic systems (Cheng et al., 2011). Aripiprazole’s partial excitatory effect on D2 receptors can lead to shifts in the dopaminergic state, either increasing or decreasing it, which might act as a hiccup trigger. Furthermore, aripiprazole’s strong affinity for D3 receptors is noteworthy. Given that dopamine agonists with high D3 affinity have been implicated in causing hiccups, it is suggested that D3 receptors might play a pivotal role in hiccup pathogenesis (Silverman et al., 2014). At lower dosages (<7.5 mg/day), aripiprazole might function as a dopamine agonist, stimulating D2 and D3 receptors at the brain stem’s “hiccup center.” Carbone et al. (2021). Furthermore, aripiprazole’s ability to amplify phrenic nerve activity could instigate the hiccup reflex arc, a process facilitated by the activation of 5-HT1A receptors and the antagonism of 5-HT2A receptors. Given these multifaceted interactions, it is conceivable that hiccups arise from an intricate imbalance across several peripheral and central neurotransmitter systems.

#### 2.3.4 Onset and resolution timing of aripiprazole-induced hiccup

A significant majority of patients (90.9%) experienced hiccups within 1–2 days following the initiation of aripiprazole. Specifically, 68.2% (15/22) of patients developed hiccups on the first day, 20.8% (5/22) between the first and second day, and 9.1% (2/22) reported onset on the fifth and sixth days. However, 7 cases did not specify the exact timing of hiccup onset and resolution related to aripiprazole administration. The duration of these hiccups varied, ranging from a brief 1–2 h to an extended 6 days. In our documented case, the patient experienced hiccups rapidly, within 6 h of aripiprazole administration. Considering aripiprazole’s half-life 4,500 min and metabolite 5,640 min, most patients observed hiccup cessation between one to 4 days post-drug discontinuation. This included instances where hiccups subsided within 6–7 h (1 case), 12 h (2 cases), 24–56 h (5 cases), beyond 4 days (1 case), and up to a week (1 case). In elderly patients, the resolution might extend to 4–7 days, potentially due to reduced metabolic clearance of the drug (Behere et al., 2007). Conversely, adolescents, with their faster metabolic rates (Li et al., 2022), might experience quicker resolution.

Given these findings, it is crucial for clinicians to closely monitor potential side effects, especially hiccups, during the initial 1–2 days of aripiprazole therapy.

#### 2.3.5 Approach to the problem

The predominant strategy for alleviating aripiprazole-induced hiccups was discontinuation of the drug, which proved effective in 51.7% (15/29) of cases. When aripiprazole was co-administered with benzodiazepines, halting the benzodiazepines emerged as the favored approach, resolving the issue in 24.1% (7/29) of cases. Additionally, gabapentin/pregabalin served as a viable remedy, benefiting 20.7% (6/29) of patients.In the majority of cases, hiccups subsided once aripiprazole administration ceased. Transitioning from aripiprazole to other antipsychotics, such as risperidone, quetiapine, olanzapine, acenapine, or clozapine, prevented the recurrence of hiccups in 41.3% (12/29) of cases. For some, allowing the hiccups to naturally diminish over time was the chosen route, with 6.8% (2/29) of patients experiencing eventual relief. Typically, hiccups would dissipate or lessen within a week. Prior research has indicated that the 5-HT1A receptor, among other mechanisms, may play a role in mitigating hiccups. Consequently, we postulate that the observed remission of hiccups in a small patient cohort could be attributed to an increased tolerance and altered sensitivity to the 5-HT1A receptor during the pharmacological intervention. It is important to note that this inference is presently supported by limited case reports, underscoring the need for more extensive research.

In three documented cases, a transition from a potent D2 antagonist (such as olanzapine, risperidone, and chlorothiol) to aripiprazole, which acts as a partial agonist for both dopamine D2 and D3 receptors, was observed to precipitate hiccups. The use of a strong D2 antagonist might lead to an upregulation of D2 postsynaptic receptors. As a result, the subsequent hiccups observed with aripiprazole could be attributed to the drug’s stimulatory effect on these upregulated D2 receptors. (Yeh, 2011; Silverman et al., 2014). Furthermore, aripiprazole’s agonistic activity on the 5HT(1A) receptor and antagonistic effect on the 5HT(2A) receptor might amplify the phrenic nerve activity, a known trigger for hiccups (Yeh, 2011).

According to the literature review, gabapentin has been identified as an effective treatment for aripiprazole-induced hiccups (Carbone et al., 2021). In contrast, other commonly used medications may not yield the desired results (Steger et al., 2015). In initial treatments for aripiprazole-induced hiccups in 5 patients (representing 17.2%, 5/29 of the cases), conventional medications proved ineffective. Specifically:One patient was administered mosapride without relief.One patient received an injection of metoclopramide to no avail. Another patient was prescribed 15 mg of lansoprazole, which did not alleviate the symptoms. Two patients were given injections of metoclopramide, but the hiccups persisted.One patient was treated with a dosage ranging from 50 to 125 mg/d of chlorpromazine, which did not prove effective. Two patients attempted physical interventions, such as holding their breath or pinching their nose while drinking, but these methods did not resolve the hiccups.

In this case study, treatments with anisodamine, baclofen, and metoclopramide yielded no discernible benefits. However, 26 h following drug cessation, the patient experienced a gradual alleviation of symptoms and managed intermittent sleep after receiving a 25 mg injection of chlorpromazine. The hiccups ceased entirely 33 h post-aripiprazole discontinuation and 7 h after the chlorpromazine administration. Thus, the resolution of the hiccups in our patient might be primarily attributed to the progressive elimination of aripiprazole from his system. Chlorpromazine, typically dosed at 25–50 mg tid, has been documented as an effective remedy for intractable hiccups (Steger et al., 2015). This drug has been approved by FDA for the treatment of hiccups. However, due to concerns about long-term neurological effects, including hypotension and drowsiness, there are inconsisitencies in the treatment consensus among different countries (Petroianu and Lorke, 2020). Notably, only one case in the literature reported the ineffectiveness of chlorpromazine in treating aripiprazole-induced hiccups.

Interestingly, out of five cases, three experienced a recurrence of hiccups upon re-administration of aripiprazole, while the other two did not exhibit any such symptoms (Kutuk et al., 2016). Consequently, if hiccups do not significantly impair a patient’s daily activities, reintroducing aripiprazole might be considered, especially in the absence of superior alternatives. Over time, as patients build tolerance, the recurrence of hiccups may diminish.

#### 2.3.6 Drug combination risks

##### 2.3.6.1 Benzodiazepine co-administration increases hiccup risk

Out of 17 cases where aripiprazole was combined with benzodiazepines (Caloro et al., 2016; Chen et al., 2018), hiccups subsided in 7 cases following aripiprazole withdrawal, in 6 cases after discontinuing benzodiazepine, and in 3 cases upon ceasing both medications (De Filippis et al., 2015). In another instance, diloazepam (2 mg/d) was switched to diazepam (12 mg/d) to address the hiccups, but this proved ineffective. The patient discussed in our report was also on a combined regimen of aripiprazole and benzodiazepine. Given his pronounced anxiety, and the importance of benzodiazepine as an anxiolytic, its dosage was not reduced post-hiccup onset. Due to the significant impact of the symptom on sleep and emotional wellbeing, our chosen intervention was to discontinue aripiprazole.

Previous research has identified that the “reflex arc” associated with hiccups can be activated by the binding of the GABA(a) receptor to voltage-calcium ions. Benzodiazepines can enhance the activation of GABA(a), potentially leading to hiccups. A retrospective study revealed a staggering 70-fold increase in hiccup risk among patients who consumed both aripiprazole and benzodiazepines compared to those who did not. Intriguingly, this risk was even more pronounced in male patients. There appears to be a dose-dependent inverse relationship between benzodiazepines and hiccups (De Filippis et al., 2015). At lower doses, benzodiazepines have been linked to hiccup onset (Caloro et al., 2016). Conversely, at higher doses, they might serve as an effective treatment for hiccups (Carbone et al., 2021). Our literature review suggests that a common strategy for managing aripiprazole-induced hiccups involves either reducing the dosage or discontinuing benzodiazepines.

##### 2.3.6.2 Drug interactions influencing aripiprazole metabolism can elevate hiccup risk

Our literature review identified a case of hiccups resulting from the combined use of aripiprazole and methylphenidate, and another case from the combination of aripiprazole and sertraline. Aripiprazole’s elimination is linked to two cytochrome P450 enzymes: CYP2D6 and CYP3A4. Methylphenidate acts both as a substrate for CYP2D6 and a weak inhibitor of the same enzyme. By either obstructing aripiprazole’s metabolism or competing for CYP2D6 metabolism, methylphenidate might increase aripiprazole’s blood concentrations (Kutuk et al., 2017). Similarly, sertraline impedes the CYP2D6 enzyme’s degradation of aripiprazole (Li et al., 2022). Consequently, drugs that inhibit P450 enzymes (specifically CYP2D6 and CYP3A4) can extend aripiprazole’s half-life, heightening the central nervous system’s risk of hiccup induction. Additionally, both aripiprazole and methylphenidate enhance dopaminergic activity, which may further contribute to the heightened risk of hiccups when used in combination (Petroianu and Lorke, 2020).

##### 2.3.6.3 Antiviral drug

Tenofovir fumarate is commonly associated with adverse reactions such as nausea, vomiting, abdominal distension, and hiccups (Marcellin et al., 2019). In the case we examined, the patient did not experience hiccups despite long-term use of this medication. However, it remains to be determined whether the concurrent use of aripiprazole might elevate the risk of hiccups. Further research is warranted.

### 2.4 Influence of comorbid conditions

A review of the literature revealed that five cases had a history of head trauma (Serafini et al., 2019). While this data is not conclusive enough to establish a direct link between aripiprazole-induced hiccups and a prior history of head injury, it is a phenomenon that warrants further scrutiny.

Hiccups are commonly associated with hyponatremia. A decrease in sodium levels by 103 mEq/L can amplify the risk of hiccups by 17-fold. Psychotropic medications pose the highest risk of hyponatremia during the initial 2 weeks of treatment, irrespective of the dosage. Aripiprazole has been linked to hyponatremia (Behere et al., 2007). However, only one case in the literature review mentioned hiccups associated with aripiprazole-induced hyponatremia. Our patient maintained normal sodium levels. Thus, the precise relationship between aripiprazole, hiccups, and hyponatremia remains ambiguous.

### 2.5 Psychosocial factors

Interestingly, our patient experienced a brief recurrence of hiccups during follow-up after discontinuing aripiprazole. These hiccups were alleviated through suggestive therapy, potentially linked to his anxious and suggestible personality traits. Psychogenic hiccups have been observed in psychiatric conditions such as conversion disorder, somatic symptom disorder, illness anxiety disorder, and anorexia nervosa. Potential treatments encompass psychotherapy, hypnotherapy, anti-anxiety therapy, and antipsychotic therapy (Theohar and McKegney, 1970). The dual occurrences of hiccups in this case may stem from distinct mechanisms, necessitating careful differentiation in clinical practice. It's also plausible that the patient’s personality traits heightened his self-awareness of the aripiprazole-induced hiccups, intensifying his distress. Thus, while drug-induced hiccups are not currently linked to a specific condition, assessing a patient’s psychosocial factors remains crucial in management.

## 3 Conclusion

In summary, hiccups induced by aripiprazole predominantly affect adolescent and middle-aged male patients, accounting for 86.7% of cases. There’s no discernible link between the underlying mental disorder diagnosis and the dosage of aripiprazole. Most patients (90.9%) experience hiccups within 1–2 days of starting aripiprazole, with symptoms typically resolving within 1–4 days of discontinuation. The most prevalent intervention for these hiccups is the cessation of aripiprazole, effective in 51.7% of cases. When aripiprazole is co-administered with benzodiazepines, discontinuing the latter proves beneficial in 24.1% of cases. Additionally, treatments with Gabapentin or pregabalin have been effective in 20.7% of cases. The combination of aripiprazole and benzodiazepines emerges as a significant risk factor for inducing hiccups. Clinicians should be vigilant about this potential side effect, especially during the initial stages of treatment. Moreover, a comprehensive personality and psychological assessment is paramount in managing hiccups in patients with psychiatric conditions. This knowledge aids in refining clinical treatment strategies and in the early identification and mitigation of adverse drug reactions.

## Data Availability

The raw data supporting the conclusion of this article will be made available by the authors, without undue reservation.
